# A Great Man

**Published:** 1892-07

**Authors:** Owen Meredith


### A GREAT MAN.

That man is great, and he alone,
Who serves a greatness not his own,
For neither praise nor pelf;
Content to know and be unknown,
Whole in himself.

Strong is that man, he only strong,
To whose well-ordered will belong,
For service and delight,
All powers that in face of wrong
Establish right.

And free is he, and only he,
Who, from his tyrant passions free,
By fortune undismayed,
Has power upon himself to be
By himself obeyed.

If such a man there be, where'er
Beneath the sun and moon he fare,
He cannot fare amiss;
Great Nature hath him in her care,
Her cause is his.—*Owen Meredith.*

